# Oxidative Stress in Drug-Induced Liver Injury (DILI): From Mechanisms to Biomarkers for Use in Clinical Practice

**DOI:** 10.3390/antiox10030390

**Published:** 2021-03-05

**Authors:** Marina Villanueva-Paz, Laura Morán, Nuria López-Alcántara, Cristiana Freixo, Raúl J. Andrade, M Isabel Lucena, Francisco Javier Cubero

**Affiliations:** 1 Unidad de Gestión Clínica de Gastroenterología, Servicio de Farmacología Clínica, Instituto de Investigación Biomédica de Málaga-IBIMA, Hospital Universitario Virgen de la Victoria, Universidad de Málaga, CIBERehd, 29071 Málaga, Spain; marvp75@gmail.com (M.V.-P.); lucena@uma.es (M.I.L.); 2 Department of Immunology, Ophthalmology and ENT, Complutense University School of Medicine, 28040 Madrid, Spain; lmoran@ucm.es (L.M.); nurial04@ucm.es (N.L.-A.); 3 Health Research Institute Gregorio Marañón (IiSGM), 28009 Madrid, Spain; 4 CINTESIS, Center for Health Technology and Services Research, do Porto University School of Medicine, 4200-319 Porto, Portugal; cristiana.tfreixo@gmail.com; 5 12 de Octubre Health Research Institute (imas12), 28041 Madrid, Spain

**Keywords:** DILI, oxidative stress, risk factors, biomarkers, mechanisms

## Abstract

Idiosyncratic drug-induced liver injury (DILI) is a type of hepatic injury caused by an uncommon drug adverse reaction that can develop to conditions spanning from asymptomatic liver laboratory abnormalities to acute liver failure (ALF) and death. The cellular and molecular mechanisms involved in DILI are poorly understood. Hepatocyte damage can be caused by the metabolic activation of chemically active intermediate metabolites that covalently bind to macromolecules (e.g., proteins, DNA), forming protein adducts—neoantigens—that lead to the generation of oxidative stress, mitochondrial dysfunction, and endoplasmic reticulum (ER) stress, which can eventually lead to cell death. In parallel, damage-associated molecular patterns (DAMPs) stimulate the immune response, whereby inflammasomes play a pivotal role, and neoantigen presentation on specific human leukocyte antigen (HLA) molecules trigger the adaptive immune response. A wide array of antioxidant mechanisms exists to counterbalance the effect of oxidants, including glutathione (GSH), superoxide dismutase (SOD), catalase, and glutathione peroxidase (GPX), which are pivotal in detoxification. These get compromised during DILI, triggering an imbalance between oxidants and antioxidants defense systems, generating oxidative stress. As a result of exacerbated oxidative stress, several danger signals, including mitochondrial damage, cell death, and inflammatory markers, and microRNAs (miRNAs) related to extracellular vesicles (EVs) have already been reported as mechanistic biomarkers. Here, the status quo and the future directions in DILI are thoroughly discussed, with a special focus on the role of oxidative stress and the development of new biomarkers.

## 1. Introduction

Drug-induced liver injury (DILI) is an adverse reaction caused by exposure to drugs and herbal medicines or other xenobiotics. Depending on the presumed mechanism of action of the causative drug, DILI is typically classified as intrinsic (direct) or idiosyncratic [1], although indirect injury is emerging as a third type [2]. Intrinsic DILI is related to the cytotoxic properties of the causative drug or its metabolite(s). In this case, liver injury is dose-dependent and predictable, and damage can be reproduced in animal models [3]. Acetaminophen (APAP) toxicity is the most common cause for this type of DILI [4,5,6,7]. In contrast, idiosyncratic DILI**** is mostly host-dependent, multifactorial and unpredictable, since it is determined by both the properties of the drug and its interaction with environmental and host factors [8]. Idiosyncratic DILI is usually not dose-dependent, although the exposure to a threshold dose in each susceptible individual is necessary [9,10]. Moreover, the delay between starting the drug and the onset of clinical signs of liver injury is another characteristic of idiosyncratic DILI. Indirect liver injury is caused by an indirect action of the drug on liver or immune system, and can induce a new liver condition or exacerbate a preexisting one, such as worsening of hepatitis B or C.

The lines that distinguish types of hepatotoxicity are blurred, and majority of drug-induced liver reactions are considered idiosyncratic. Indeed, this is an unresolved issue and rather an academic classification as research over the last years has demonstrated that there are host susceptibility factors that influence the risk of intrinsic damage and, on the contrary, for drugs that are believed to cause idiosyncratic liver damage, there might be a dose threshold. Therefore, unless stated otherwise, the term DILI is used for idiosyncratic drug-induced hepatotoxicity in this review.

Due to its unpredictability and poorly understood pathophysiology, DILI is considered an exclusion diagnosis and, therefore, a diagnostic challenge [11,12].

### 1.1. Epidemiology

The true incidence of DILI is difficult to establish due to the rarity of the condition and the lack of homogenous diagnostic criteria across studies [13]. In retrospective studies based on medical records from patients of the Sahlgrenska University Hospital (Sweden) and the UK-based General Practice Research Database, DILI annual incidence was established to be 2.3 and 2.4 cases per 100,000 inhabitants, respectively [14,15]; while in population-based prospective studies in France and Iceland, DILI annual incidence was 13.9 and 19.1 cases per 100,000 inhabitants, respectively [16,17]. Due to the high frequency of polymedication, demographic changes with a growing aging population, and the increasing consumption of herbal and dietary supplements (HDS), DILI incidence is expected to rise in the future. Interestingly, in western countries, DILI is mainly caused by pharmacological drugs, contrary to Asian countries, where herbal products and Traditional Chinese Medicines predominate [18]. However, herbal-induced liver injury (HILI) is increasing worldwide due to the rise in the use of herbal supplements and remedies, representing a major health problem [19]. Still, hepatotoxicity of HDS is particularly difficult to demonstrate due to the difficulty in determining the toxic(s) compound(s) and the frequently hidden self-medication [20].

DILI can be caused by a wide variety of drugs, including antibiotics, cardiovascular drugs, central nervous system (CNS) agents, nonsteroidal anti-inflammatory drugs (NSAIDs), and others, such as HDS (Table 1). Due to the low frequency of DILI and the heterogeneity of presentation as it may mimic any other acute or chronic liver disorder, it is usually recognized at post-marketing, being a reason for drug withdrawal and the adoption of regulatory measures, bearing a high impact on drug development, regulatory bodies and physicians and patients alike [21].

### 1.2. Diagnosis

The clinical presentation of DILI is very variable in severity and phenotypic expression, ranging from asymptomatic elevation of liver enzymes to acute liver failure (ALF). Other more common causes of hepatic injury must be evaluated and excluded first. DILI diagnosis is currently based on a comprehensive clinical history, detailed drug exposure investigation, exclusion of common causes of hepatic injury according to clinical context and pattern of laboratory liver abnormalities and the application of clinical assessment scales to determine the likelihood of the reaction. Therefore, physician’s suspicion of DILI is a first step in approaching this challenging diagnosis [23].

Clinically, DILI patients present an extensive range of unspecific symptoms, which can include fatigue, nausea, abdominal pain, pruritus and jaundice, that are frequent in other liver diseases. If the clinician has a suspicion of DILI, after detection of liver biochemistry abnormalities, a detailed interview with the patient is essential to obtain information about the exposure to prescription and non-prescription drugs, HDS, as well as latency (time from drug initiation to liver damage detection) and effect of suspected causative agent withdrawal [21]. In fact, withdrawal of the offending medication is critical in DILI suspicion and management.

An international consensus group and the European Association for the Study of the Liver (EASL) have proposed some threshold criteria for definition of a case as being DILI [3,23,24]: Serum alanine aminotransferase (ALT) elevation ≥ 5 times the upper limit of normal (ULN), serum alkaline phosphatase (ALP) ≥ 2 × ULN or the combination of ALT ≥ 3 × ULN with simultaneous elevation of total bilirubin (TBL) exceeding 2 × ULN.

Since liver biopsy is not usually performed for DILI diagnosis, liver biochemistry is used to define the type of liver damage. The different laboratory patterns can be determined by the R-ratio of serum markers of liver injury (ALT/ALP) at DILI presentation [24]. It is considered hepatocellular when ALT ≥ 5 × ULN alone or when R-ratio is ≥5, and cholestatic when ALP ≥ 2 × ULN alone or if R-ratio is ≤2. Mixed liver injury is considered when ALT ≥ 3 × ULN, ALP ≥ 2 × ULN, and R-ratio is 2–5 [25]. Hepatocellular injury is characterized by an elevation of serum transaminases related to hepatocyte damage triggered by the toxin, and it is more likely to be associated with a poor outcome. Cholestatic injury is manifested by increased levels of ALP, γ-glutamyl transpeptidase (γ-GT), and conjugated bilirubin in serum [26], suggesting impaired bile flow regulation leading to bile deposition in the liver. It is associated with not negligible mortality. Meanwhile, mixed liver injury has the lowest mortality rate [23]. Several cohort studies show that acute hepatocellular hepatitis is the most common manifestation of DILI [14,25,26].

Cholestatic injury is associated with pruritus and asthenia most frequently than other phenotypes. Some DILI patients also show immunoallergic features and/or skin reactions (i.e., cutaneous rash), which seems to have more severe outcomes [27,28,29]. For example, drugs such as carbamazepine, phenytoin, and dapsone are associated with liver injury accompanied with cutaneous hypersensitivity features [30,31]. In addition, DILI can present with other phenotypes [23] as drug-induced autoimmune hepatitis (DILI-AIH), which involves hepatocellular liver damage with features of idiopathic AIH or drug-induced fatty liver disease, which is characterized by an accumulation of lipids in hepatocytes (steatosis).

After the exclusion of a potential infinite list of alternative explanations, the application of the RUCAM (Roussel Uclaf Causality Assessment Method)/CIOMS (Council for International Organizations of Medical Sciences) score is used to translate the DILI suspicion into categories of probability. RUCAM provides a sum score that ranges from −5 to +14 points, while evaluating seven domains (time to onset of the reaction from both the beginning and cessation of use of the causative agents; course of the reaction; risk factors; concomitant medications; non-medication related causes; previous information on the medication and response to re-administration). According to the final score obtained, the hepatic reaction is classified into five categories of probability: highly probable (>8), probable (6–8), possible (3–5), unlikely (1–2), or excluded [32,33].

Currently, no reliable in vitro test exists to support the diagnosis of DILI. Recently, an in vitro assay to identify the drug responsible for causing DILI using monocyte-derived hepatocyte-like (MH) cells from patients with DILI suspicion has been proposed [34]. These cells are derived from peripheral monocytes and retain several of their characteristics, showing inducible activities of different cytochrome P450 (CYP450) enzymes, reflecting the activities in primary human hepatocytes of the individual [35]. However, this method awaits external validation.

## 2. Potential Mechanisms Involved in DILI Pathogenesis

The liver plays an important role in metabolizing drugs or exogenous toxicants, protecting the organism from potential toxic chemicals [36]. Bioactivation processes of parent drugs rendering reactive metabolites and the mechanisms involved in detoxification and excretion of xenobiotics (most of them under genetic control) are critical for understanding the mechanisms of DILI [37]. However, many different hypotheses have been proposed due to the multivariant nature of the disease [38].

### 2.1. Drug Factors

Physicochemical and toxicological drug properties affect DILI risk [39], contributing to initial cell damage that induces an adaptive and innate immune response. First, currently there is a consensus about the necessity of drug/metabolite exposure to a specific threshold level to DILI be initiated [8]. In fact, there is an association between daily dose of a drug and poor DILI outcome [10].

Drug lipophilicity is also associated with DILI risk, since it can enhance drug uptake from blood into hepatocytes, which results in an accumulation of reactive metabolites. In fact, a lipophilicity of LogP ≥ 3 in combination with high daily dose of drug (≥100 mg) are associated with severe DILI [40].

Finally, the potential of a drug to form reactive metabolites is also associated with the pathogenesis of DILI [41], due to their own toxicity and their ability to form drug-endogenous proteins adducts, which can activate the immune response [42]. However, drugs unknown to form reactive metabolites, such as ambrisentan, flecainide, maraviroc, or bosentan can also cause DILI [43].

### 2.2. Metabolic Mechanisms

**Figure 1:**
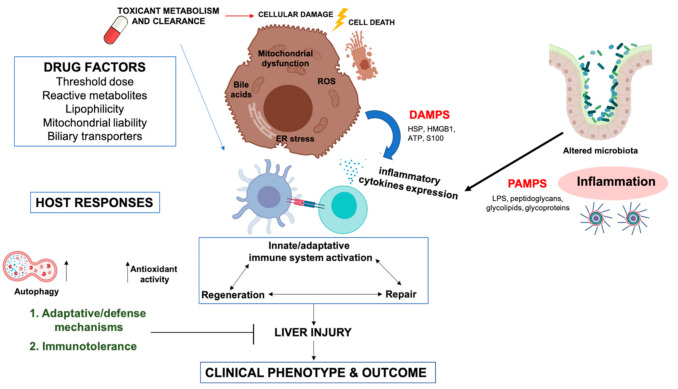
Cellular and molecular mechanisms involved in idiosyncratic drug-induced liver injury. Two key players in DILI, drug and host factors may interact in a multi-faceted manner at different functional pathways and determine individual susceptibility, clinical phenotype, and outcome. The hepatocyte damage caused by the action of drugs induce a complex, multivariant host response. First, cellular damage (oxidative stress, mitochondrial dysfunction, endoplasmic reticulum (ER) stress, and bile salt export pump (BSEP) inhibition, between others) can lead to cell death, provoking cell swelling and eventually rupture of the cell membrane, with the release of intracellular content, including damage-associated molecular patterns (damage-associated molecular patterns (DAMPs), such as high mobility group box protein 1 (HMGB1), heat shock proteins (HSP), ATP, S100 proteins, etc.) which stimulate a strong inflammatory/immune response. Inflammasome has also a very important role in development of liver injury, inducing cytokines secretion to attract and activate macrophages and neutrophils. Moreover, drugs can also alter intestinal microbiota (dysbiosis), and increase intestinal permeability, releasing bacterial products (Pathogen-associated molecular patterns, [PAMPs]) into the bloodstream. PAMPs (bacterial lipopolysaccharides (LPS), endotoxins, flagellin, etc.) act as costimulatory signals for the innate immune system activation. DAMPs and PAMPs are able to bind to TLR of the innate immune cells potentiating the immune response, cytokine release, and immune cell recruitment. Furthermore, drugs can form drug-endogenous proteins adducts that can act as neoantigens. Neoantigens presentation on specific HLA molecules could cause an adaptive immune response. Some HLA polymorphisms favor the presentation of drug-adducted neoantigens. Thus, individuals carrying the HLA variant are more susceptible to develop an adaptive immune response, typically leading to a T cell response directed at hepatocytes and usually involving cytotoxic CD8 T cells that target the peptide drug exposed on MHC class I molecules on the hepatocytes. Cellular damage also induces host adaptive and defense mechanisms, such as autophagy, antioxidant response and tissue repair. Moreover, because of its biological role with constant exposure to foreign antigens, the liver has a strong natural predisposition towards immune tolerance. This tolerance prevents a substantial immune response in the presence of the chemical insult, causing, at most, a mild liver injury that resolves spontaneously despite continued drug intake (i.e., adaptation). Clinically relevant liver injury is believed to result from a breakdown in hepatic immune tolerance. Concomitant inflammation can change the cytokine environment in favor of an immune response. Host factors such as age, gender, genetic factors, lifestyles, disease conditions, and co-medications are involved in the susceptibility of significant liver damage.

Hepatocyte exposure to increased cellular stress is assumed to be the initial step in DILI development. Initial cell damage is induced by drugs and/or their reactive metabolites via covalent binding or direct damage to mitochondria, which leads to oxidative stress and the activation of stress-sensing signaling pathways, impairment of the mitochondrial function, and endoplasmic reticulum (ER) stress (Figure 1).

The mechanisms involved in the detoxification of drugs are critical in understanding the different processes triggered during DILI. The human CYP450 are membrane-bound proteins located in either the mitochondrial inner membrane or the smooth endoplasmic reticulum of hepatocytes, where they are responsible for the oxidation, peroxidation, and reduction being necessary for drug metabolism (see Section 3.4.1). The reactive metabolites generated during the metabolism of drugs are the main responsible for the sharp increase in oxidative stress directly generated in mitochondria of the injured hepatocyte [13]. Increased reactive oxygen species (ROS) can directly damage DNA, proteins, enzymes, and lipids in cells and tissues and induce immune-mediated liver damage. Some drugs (e.g., valproic acid, [VPA]) can induce enhanced generation of ROS and triggering c-Jun N-terminal kinase (JNK). Thus, leading to hepatocyte death [32]. This is a biphasic process: the early stage involves glycogen synthase kinase-3β (GSK-3β) activating mixed-lineage kinase-3 (MLK3), whilst the late phase is mediated by apoptosis signal-regulating kinase-1 (ASK1), thereby activating JNK [44], which translocates to the mitochondria and triggers hepatocyte death, resulting in the amplification of mitochondrial ROS, such as in APAP-derived toxicity [45].

Furthermore, damage-associated molecular patterns (DAMPs) released from injured hepatocytes activate innate immune responses, including cytokines such as tumor necrosis factor-alpha (TNFɑ), Fas ligand (FasL) or TNF-related apoptosis-inducing ligand (TRAIL)-expressing natural killer or natural killer T cells and neutrophils, which can activate death receptors such as TNF-R, FasR, and DR5. Besides, the activation of the necrotic cell death pathway is also present in DILI [46]. Necrosis involves cell swelling, membrane bleb formation, and eventually the rupture of plasma membrane, causing the release of cellular components from necrotic cells that elicit an inflammatory response. Alternative mechanisms of regulated necrosis have emerged in recent years, such as necroptosis, pyroptosis, and ferroptosis. However, its relevance to acute or chronic DILI needs further research. Consequently, in DILI, altered cell functioning causes exacerbated ROS that further produce loss of hepatocyte function, and damaged hepatocytes release ROS, increasing overall oxidative stress, and ultimately leading to the activation of apoptotic and necrotic pathways [46].

Metabolic activation of a drug and the generation of a reactive intermediate, the inadequate detoxification of the reactive intermediate, and the covalent binding to macromolecules can lead to subsequent liver toxicity [47]. The formation of reactive metabolites has been well documented for drugs that have been withdrawn from the market or have a warning for hepatotoxicity. Examples are nefazodone, metabolized via CYP3A4 producing hydroxynefazodone, triazoledione, and m-chlorophenylpiperazine as metabolites or VPA, whose toxicity may be related to its metabolism through CYP2C9 and CYP2A6 to 4-ene-VPA [47].

The metabolic activation of APAP is perhaps the best-documented case. Although APAP is metabolized to its glucuronidated and sulphated non-toxic metabolites in the liver, APAP overdose saturates these pathways, and the excess APAP is metabolized by CYP2E1 into the reactive metabolite N-acetyl-p-benzoquinoneimine (NAPQI), which is rapidly conjugated with glutathione (GSH), resulting in non-toxic mercapturic acid and cysteine conjugates that are excreted in the urine. When hepatic GSH levels are limited, free unconjugated NAPQI reacts with sulfhydryl groups on cysteine and lysine residues, generating NAPQI-protein adducts (APAP-protein adducts) in hepatocytes, particularly in mitochondria, leading to mitochondrial dysfunction [48] and cell death.

Generally, the elevated level of chemically reactive intermediates overwhelms the capacity of the detoxification enzymes and endogenous antioxidants. A wide variety of antioxidant mechanisms to counterbalance pro- and antioxidant compounds, including GSH, superoxide dismutase (SOD), catalase, and glutathione peroxidase (GPX) that are pivotal in detoxification, are compromised, triggering imbalance between oxidants and antioxidants, and generating oxidative stress. Most importantly, mitochondria are the most affected hepatocyte organelles by GSH deficiency [49].

#### 2.2.1. Oxidative Stress

Oxidative stress is the result of the generation of ROS, which are a by-product of normal metabolism and have significant roles in cellular signaling and homeostasis. Some DILI-causing drugs increase ROS accumulation through a variety of mechanisms [50]. Moreover, during the pathogenesis of DILI, the effect produced in the liver caused by the depletion of GSH is translated into an increase in mitochondrial H_2_O_2_ [50].

Lipid peroxidation (LPO) may be involved in the mechanism of cell death during DILI. During LPO, the generation of lipid radicals leads to the destruction of polyunsaturated fatty acids (PUFAs) in lipid membranes [47]. LPO can cause rapid catastrophic breakdown of the membrane potential and ion gradients, leading to necrotic cell death. Wendel and collaborators [51] injected APAP in mice and observed massive LPO four hours after injection, which was prevented by vitamin E pre-treatment. In principle, these results indicated a major role for LPO in APAP-mediated DILI. However, the authors fed the animals with a vitamin E-deficient diet with a high content of PUFAs. Nonetheless, a more recent study by Jaeschke’s group [52] suggested that animals on a regular diet have enough lipid-soluble antioxidants to prevent extensive LPO. APAP overdose causes severe liver damage but a minor increase in the levels of LPO in normal animals [50]. Cell injury induced by LPO requires not only oxidant formation but also impairment of the antioxidant defense systems. Altogether, LPO might not be a relevant injury mechanism in APAP-induced liver injury [53], which is the most extensively used experimental model to study DILI. Thus, the role of LPO in DILI still remains currently controversial.

But is LPO a player of DILI in other DILI-causing drugs? Two anti-arrhythmic drugs—dronedarone and amiodarone—triggered accumulation of ROS and intracellular lipids in vivo [54]. Moreover, in vivo administration of methotrexate (MTX), a folate antagonist used in the treatment of malignancies and autoimmune diseases, caused LPO product malondialdehyde (MDA) [55]. Finally, VPA treatment induced significant increase in LPO in isolated rat hepatocytes [56] and patients [57]. Thus, further investigation is needed in order to elucidate whether LPO is a consequence of tissue injury or a major cause in the mechanism of DILI.

Superoxide radicals (O_2_^•−^) can also react with nitric oxide (NO), whose generation is increased by an up-regulation of inducible NO synthase (iNOS) and endothelial nitric oxide synthase (eNOS), forming peroxynitrite (ONOO^−^). Since the O_2_^•−^ anion scarcely passes through the hepatocyte cell membrane, this process occurs exclusively within the mitochondria. The highly reactive and potent oxidant ONOO^−^ also causes nitration of protein tyrosine residues which induces damage to mitochondrial DNA and the opening of the mitochondrial membrane pore [58].

Therefore, the dysregulation of redox balance causes an impact on cellular and mitochondrial damage. The Kelch-like ECH-associated protein 1 (Keap1)/nuclear erythroid factor type 2 (Nrf2) antioxidant system is involved in the regulation of oxidative stress [59]. In normal conditions, the activity of Nrf2 is suppressed by Keap1 in the cytoplasm. Keap1 contains thiols, which bind with Nrf2. Under pathological conditions, it is produced the oxidation of these thiols causing the subsequent translocation of Nrf2 into the nucleus and promoting the antioxidant response element (ARE). This system controls the expression of antioxidant enzymes involved in the detoxification including, heme oxigenase-1 (HO-1), glutathione S-transferase (GST), glutamate-cysteine ligase catalytic subunit (GCLC), and NAD(P)H quinone oxidoreductase 1 (NQO1).

Therefore, oxidative stress imbalance (excessive generation of ROS and/or the inhibition of detoxification pathways) may be involved in DILI susceptibility and severity [60,61], since it affects mitochondrial function and DNA integrity between others, inducing cell death and immune-mediated liver damage.

Drugs associated with DILI such as troglitazone, flutamide, nimesulide, VPA, and diclofenac have been observed to increase intracellular oxidants [8]. Based on this evidence, different antioxidant compounds have been evaluated in clinical trials to find beneficial effects on DILI prevention and/or management [62]. Thus, a prospective controlled trial conducted in 50 patients concluded that the administration of N-acetylcysteine (NAC) improved survival in patients with fulminant hepatic failure after APAP overdose [63]. Currently, NAC has the Food and Drug Administration (FDA) approval for the treatment of potentially hepatotoxic doses of paracetamol and is the mainstay of therapy for APAP toxicity. Interestingly, a more recent multicenter prospective study involving 173 patients with ALF of various etiologies, including DILI-related ALF, showed that NAC improved transplant-free survival in patients at early stage non-APAP-related ALF [64], and Baniasiadi et al. conducted a randomized clinical on 60 TB patients and also found that NAC protected against anti-tuberculosis (TB) drug-induced hepatotoxicity [65].

Other compounds exhibiting antioxidant properties have received growing attention in the last years, and different randomized clinical trials have been conducted to assess the efficacy of silymarin [66,67,68,69,70,71,72,73], bicyclol [74,75,76,77], magnesium isoglycyrrhinate (MgIG) [78,79,80], tiopronin [81], and L-carnitine [82] in the prevention and/or management of DILI. Among these studies, it is worth highlighting the promising results in the reduction of DILI risk and management of the disease that bicyclol and MgIG treatments have shown. However, antioxidants effects should be interpreted cautiously given the low number of trials, the small sample sizes and the differences regarding DILI criteria between trials. Thus, more high-quality clinical trials are needed.

Interestingly, Koido and colleagues recently reported a polygenic risk score (PRS) associated with oxidative stress imbalance in the susceptibility of DILI patients to drugs including fasiglifam, amoxicillin-clavulanate, or flucloxacillin [83].

#### 2.2.2. Mitochondrial Dysfunction

The redox control in the mitochondria is essential for the normal hepatocyte function. Mitochondria have been considered an important target in DILI [84], since inhibition of mitochondrial electron transport chain (ETC) associated with oxidative phosphorylation (OXPHOS) results in ATP depletion and accumulation of ROS, leading to activation of cell death signaling pathways.

There are several reports demonstrating mitochondrial impairment triggered by different drugs known to cause DILI, including tolcapone [85], troglitazone [86,87], nefazodone [88], nimesulide [89], and cerivastatin [90,91]. Moreover, Long and colleagues used a multiscale, mechanistic model of DILI (DILIsym^®^, DILIsym Services Inc., a Simulations Plus Company, Research Triangle Park, NC, USA) to test the hypothesis that mitochondrial dysfunction was the primary mechanism underlying tolcapone-mediated toxicity, confirming that this drug had a mitochondrial uncoupling effect responsible for its hepatotoxicity [92].

In addition, troglitazone and nimesulide caused mitochondrial oxidative stress and changes in the mitochondrial permeability transition (MPT) in different in vitro models, supporting the mitochondrial dysfunction hypothesis [93,94,95,96]. The onset of the MPT to many mitochondria within a cell is known to drive a cascade of events, which leads gradually to autophagy, apoptosis, and necrotic cell death [97]. Moreover, Cho and colleagues described that the combination of rotenone (inhibitor of mitochondrial complex I) and isoniazid (IHN), an anti-TB drug associated with DILI (inhibitor of mitochondrial complex II) was synergistic in killing mice [98], confirming that underlying inhibition of complex I can trigger IHN-induced hepatocellular injury [99]. Other mechanisms of mitochondrial injury might be involved in the development of DILI. For example, VPA induces mitochondrial damage by inhibiting fatty acid metabolism, and mutations in POLG (mitochondrial DNA [mtDNA] polymerase encoding gene) are a risk factor for VPA-induced DILI [100]. Moreover, there are some drugs reported to alter mtDNA homeostasis through different mechanisms including inhibition of mtDNA replication and translation [101]. For example, tacrine, ganciclovir, and diclofenac are known to trigger mtDNA damage [102,103].

However, in spite of the lack of current in vivo studies capable of demonstrating the role of the mitochondrial dysfunction as the first cause of DILI, it has been proposed that mitochondrial dysfunction is a source of DAMPs molecules that, in turn, can stimulate an immune response [104,105,106]. Moreover, rotenone capable of activating the inflammasome, which may be involved in DILI mechanisms [107].

Mitochondria are also thought to be essential in hepatotoxicity protection. Elimination of mitochondria by selective autophagy (mitophagy) could restrict necrotic areas and promote tissue regeneration, being a promising therapeutic target of DILI [108,109].

#### 2.2.3. Endoplasmic Reticulum (ER) Stress

It has been previously reported that ER stress is produced during APAP-induced liver injury due to an overaccumulation of ROS, which causes the dysregulation of Ca^2+^ balance leading to the induction of the unfolded protein response (UPR) [110]. There are three major proteins involved in this stress response; the inositol-requiring enzyme 1 (IRE1), the protein kinase RNA (PKR)-like ER kinase (PERK), and the activating transcription factor 6 (ATF6), which, in homeostatic conditions, are maintained inactivated by attaching to the binding immunoglobulin protein (BiP) [111]. However, the increase of unfolded proteins that compete with the transducers for BiP binding triggers their activation. If the programmed mechanisms in the cell cannot mitigate ER stress, cell death is triggered in an intricate mechanism that involves caspases activation, Ca^2+^ leakage from the ER and mitochondrial damage.

Animal studies yielded controversial results about the UPR response after APAP administration. While Nagy et al. observed the induction of ER stress following intraperitoneal administration of APAP [112,113], another study did not find any signs of UPR activation [114]. Recently, Uzi and colleagues [110] reported that ER stress and UPR activation are a late event in the cascade of responses activated by APAP and coincided with upregulation of CHOP, a transcriptional repressor downstream of PERK and IRE1 that activates pro-apoptotic genes.

A recent study developed by the group of Maiuri showed that the use of diclofenac was responsible for cytotoxicity in human hepatocytes due to increased levels of intracellular Ca^2+^ and the activation of the ER stress sensor PERK and JNK [115].

Altogether, it is very likely that mitochondrial damage, leading to the sustained activation of cell death signaling pathways such as JNK and the onset of ER stress, might be intertwined mechanisms, as it has been recently shown [48], adding complexity to the pathophysiology of DILI.

### 2.3. BSEP Inhibition

The canalicular bile salt export pump (BSEP) is the only hepatocellular export system for primary bile salts into the canaliculus. BSEP inhibition has been proposed as a common mechanism of drug-induced cholestasis [116,117], since a complete genetic deficiency of BSEP leads to cholestatic liver injury and liver failure [118]. Evidence suggests that some drugs, such as bosentan [119] or troglitazone [120] induce DILI by inhibition of BSEP, supporting the hypothesis that the retention of bile acids in hepatocytes can induce cellular stress. On the other hand, the inhibition of BSEP or other bile salt transporters can initiate the immune response directly or through the release of DAMPs [38]. However, BSEP inhibitory potency alone is not enough for determining DILI risk, and additional factors such as mitochondrial dysfunction [121] or inhibition of multidrug resistance-associated proteins (MRP) [122] should be considered.

### 2.4. Activation of the Immune Response

It has been increasingly clear that the immune response during DILI is determinant, since the presence of T cells and immune system activation in patients with DILI have been observed [39,111,112]. The immune response consists of a hypersensitivity reaction which provokes an inflammatory response that involves the innate and the adaptive immune system. Different hypotheses have been suggested to explain drug-induced immune system activation [46].

#### 2.4.1. The Hapten Hypothesis

Haptens are small molecules that elicit an immune response only when covalent binding to endogenous proteins, forming adducts. Some drugs can form adducts by binding with endogenous proteins. When the drug-protein adduct (neoantigen) is taken up by antigen-presenting cells (APCs) and presented on major histocompatibility complex (MHC) class II proteins to T cells, it can elicit a subsequent adaptive immune response [42]. However, this hypothesis alone cannot explain why only a minority of patients developed DILI induced by drugs described to form haptens. Moreover, flucloxacillin, a well-known hepatotoxicant, was found to have a hapten-dependent and a hapten-independent mechanism of adaptive immune activation, suggesting additional immunological pathways [123].

#### 2.4.2. The Danger Hypothesis

This hypothesis complements the hapten hypothesis and supports that, in order to precipitate an adaptive immune response, it is required an associated damage, a “danger signal” [124]. This signal may include any intrahepatic or extrahepatic stressors, including ROS, mild inflammation or infection [125].

After cell damage and cell death pathway activation, antigens derived from DAMPs are released, binding to their respective pattern-recognition receptors (PRRs) on the APCs. Depending on the endocytic pathway and the nature of the antigen, the APCs will present it to the T cells on MHC I or MHC II molecules stimulating the adaptive immune response [126]. Besides, the danger hypothesis involves the costimulation of T cells by APCs through B7 receptors (CD80 and CD86) binding to CD28 on T cells. This costimulatory signal is required in order to induce an immune response instead of promoting immune tolerance [127].

Additionally, DAMPs are able to bind to Toll-like receptors (TLR) of the innate immune cells potentiating the immune response, cytokine release, and immune cell recruitment [128]. Among the main DAMPs found in the liver, the high mobility group box protein 1 (HMGB1), ATP, heat shock proteins (HSP), and S100 proteins are included [129]. Binding to these receptors triggers downstream signaling pathways leading to the activation of caspase-1 and the consequent cleavage of proinflammatory cytokines including interleukins IL-1β and IL-18 [130], FasL, interferon gamma (IFNγ), and TNFɑ [46].

#### 2.4.3. The Pharmacological Interaction (p-i) Hypothesis

This hypothesis postulates that chemically inert drugs can activate certain T cells by specifically and directly forming non-covalent interactions with MHC molecules with which they fit with a sufficient affinity, triggering the activation of the immune system [131,132,133,134]. In the last years, different drugs have been suggested to activate the immune system through p-i based stimulation [123,131,135,136,137,138].

#### 2.4.4. The Altered Peptide Repertoire Hypothesis

This hypothesis proposes that a drug can interact with MHC I molecules in a specific and non-covalent fashion and leads to presentation of altered endogenous peptides which elicit immune reactions. It can be regarded as a subset of p-i concept, but with the main key difference being the binding of novel self-peptides to the drug-MHC complex [139].

#### 2.4.5. The Multiple Determinant Hypothesis

This hypothesis suggests that DILI is dependent on the overlap of many different factors such as gender, age, genetics, environmental and physiological factors, etc. that increase the probability of an adverse hepatic event [140,141]. Therefore, unless different factors concurred simultaneously, DILI will not develop. This might partially explain why the disease is so infrequent despite the risk genetic polymorphisms being common in the population.

#### 2.4.6. The Inflammatory Stress Hypothesis

This hypothesis suggests that a potential inflammation occurring during drug treatment could interact with the action of the compound and produce liver injury [128]. Hepatic inflammation is often observed in DILI; therefore, it is suggested that DILI reactions could be unmasked by inflammation occurring during drug therapy. Inflammagens could bind to TLR or T-cell receptors (TCR), initiating the expression of inflammatory mediators. The inflammatory stress hypothesis has provided the first animal models in which liver injury is induced from different drugs associated with human DILI [142,143].

Moreover, a common mechanism of immune response involves activation of the inflammasome [144]. A recent study showed that the supernatant (presumably containing DAMPs) from the incubation of human hepatocytes with drugs that induce DILI activates the inflammasome in THP-1 cells, a macrophage cell line [145,146].

## 3. Risk Factors

### 3.1. Age

Age as a risk factor for DILI development remains unclear [147]. Data from two large prospective studies, the US Drug-Induced Liver Injury Network (DILIN) and the Spanish DILI Registry, did not find any differences in DILI distribution between older and younger participants [148,149]. However, older population showed a higher risk for cholestatic liver injury than younger people, who were associated with hepatocellular damage [148,149,150,151,152].

Nevertheless, age could be determinant for DILI induced by specific drugs [147], principally antimicrobials and cardiovascular drugs. IHN and flucloxacillin-induced liver injury risk are described to increase with age [153,154,155]. Meanwhile, valproic acid-associated hepatotoxicity is more frequent in children under 10 years old [156].

### 3.2. Gender

Influence of gender in DILI incidence is ambiguous, since a balance between male and female in DILI series has been observed [148,149,152]. However, gender may influence DILI development triggered by specific causative agents [23]. For example, female susceptibility to developing DILI with autoimmune features has been described for minocycline and nitrofurantoin-associated hepatotoxicity [157,158,159].

On the other hand, some differences in DILI outcome between genders have been detected. Female gender is associated with hepatocellular pattern of liver injury, AIH, and a worse outcome [148,149,160].

### 3.3. Alcohol Consumption

The role of alcohol consumption in DILI still generates controversy, since there is no evidence that alcohol might be associated with neither susceptibility to DILI nor worse outcomes in DILI patients [161]. However, currently alcohol use (more than two drinks per day for women and more than three drinks per day for men, calculating 10 g ethanol for each drink) is included in the RUCAM causality assessment scale as a risk factor for DILI [162], which is hardly justified at the light of the available data. However, regular alcohol intake may be a contributing factor for DILI associated with specific drugs such as IHN, MTX, and halothane [23,163].

### 3.4. Drug Metabolism Genetic Polymorphisms

As we have argued above, formation of reactive metabolites is likely to be an initiating event in DILI. High levels of reactive metabolite formation in an individual may be due to high levels or increased activities of enzymes from CYP450 family. Alternatively, individuals may have low levels or reduced activities of enzymes that detoxify reactive metabolites, such as UDP-glucuronosyltransferases (UGT), N-acetyl transferases (NAT), and GST [164,165,166,167]. Finally, levels or expression of transporter proteins would modulate excretion of the water-soluble metabolites into bile or systemic circulation, being responsible for the extension of exposure of hepatocytes to the drug/reactive metabolite [160,162,163] (Table 2). Therefore, investigations on genetic susceptibility to hepatotoxicity have been principally focused on drug metabolism, detoxification genes, and transporters [168].

#### 3.4.1. Cytochrome P450 Family

CYP450 enzymes are involved in oxidation, reduction or hydrolysis of substrates, being implicated in reactive metabolite formation. Therefore, genes of CYP450 family are an interesting target for genetic studies of DILI susceptibility.

A recent assessment of 254 drugs has shown that compounds that are substrate of CYP450 possess a higher risk of causing DILI, due to the formation of reactive metabolites [188]. Besides being a substrate, drugs and herbal supplements can also act as either inhibitors or inducers of CYP450 enzymes by affecting the pregnane X receptor (PXR) and the constitutive androstane receptor (CAR), influencing the risk of suffering DILI [189]. However, although the variants of the enzymes of the CYP2C subfamily lead to high heterogeneity in drug metabolism, different studies did not find any associations between CYP2C8, CYP2C9 and CYP2C19 polymorphisms and susceptibility to DILI due to drugs substrate of these cytochromes [189]. Nevertheless, genetic polymorphisms of CYP450 enzymes can influence susceptibility to DILI induced by specific drugs. For example, different studies have shown that genetic polymorphisms of CYP2E1 influenced susceptibility to anti-TB drug induced hepatotoxicity [189]. Moreover, genetic variations of CYP2B6 have been associated with increased risk of hepatotoxicity following ticlopidine [177] and efavirenz [174].

#### 3.4.2. UDP-Glucuronosyltransferases

UGT enzymes are responsible for the process of glucuronidation, the addition of a glucuronic acid moiety to xenobiotics in order to favor the excretion of drugs, toxins or even endogenous substances. Some variants of the enzymes of the UGT2B subfamily have been associated with DILI risk. For example, the UGT2B7*2 allele has been associated with increased risk hepatotoxicity following diclofenac [164,190].

#### 3.4.3. N-Acetyl Transferases

NAT enzymes mediate N-acetylation of a wide range of acrylamine and hydrazine substrates, being involved in detoxification of multiple reactive metabolites. Acetylhydrazine is a key IHN metabolite that contributes to DILI development induced by this anti-TB drug. It can undergo further metabolism by the enzyme NAT2 to the less toxic diacetylhydrazine. Therefore, NAT2 has been described to have an implication in IHN-induced liver injury [191]. The different alleles of NAT2 gene can be classified as fast acetylators (NAT2 activity within the normal range), slow acetylators or ultra-slow acetylators, according to their efficiency to form diacetylhydrazine. Therefore, it was hypothesized that slow acetylators may indirectly increase the accumulation of the toxic metabolite acetylhydrazine due to their slower rate of detoxification. However, the first studies about the association between acetylation status and anti-TB DILI were controversial [192,193,194,195,196,197,198,199,200,201]. Some of them observed that fast acetylators were prone to anti-TB DILI [192,193,194], others that slow acetylator status was a significant risk factor of anti-TB DILI [195,196,198,199,200], and some of them did not find any association between the acetylation status and risk of anti-TB DILI [197,201]. These studies were based on determination of NAT2 status by administration of a probe drug (phenotype analysis) rather than by direct genotyping of DNA. The phenotyped acetylator status can be often influenced by many extrinsic factors such as age, sex, alcohol consumption, comorbidities, etc. Moreover, the different study designs (e.g., rechallenge or not), the phenotyping methods used and the different combination of anti-TB drugs chosen in these studies could be responsible for the discrepancies observed.

Determination of the acetylator status by the genotype of NAT2 has alleviated some of these discrepancies. The first study involving NAT2 genotyping in patients with IHN DILI concluded that those positive for variants associated with slow acetylation showed an increased risk of disease [202]. Several subsequent studies have confirmed this finding [203,204,205,206,207,208,209,210,211].

Moreover, a very recent work by Aithal’s group showed that the genotype NAT2*6/NAT2*7 (ultra-slow acetylators) is associated with IHN-induced DILI [212].

#### 3.4.4. Glutathione-S-Transferases

GSTs are a family of phase II enzymes that catalyze the conjugation of the reduced form of GSH to xenobiotic substrates such as drugs for their detoxification. There are eight distinct classes identified in the GST family; however, their implication in DILI has been only demonstrated for GSTM1 and GSTT1. Deficiency in GST activity, because of homozygous null mutations at GSTM1 and GSTT1 loci, modulate susceptibility to drug- and xenobiotic-induced hepatotoxicity. Its prevalence is nearly 10% to 25% in European countries for null GSTT1 and ~50% for null GSTM1, while Asian countries go from 15% to 50% and 23% to 50%, respectively [213]. A genomic study of DILI patients from the Spanish Registry concluded that carriers of double GSTT1-M1 null genotypes had a 2.70-fold increased risk of developing DILI. The genetically determined reduction in the ability to detoxify electrophilic compounds might play a role in determining the susceptibility to develop DILI, as a general mechanism that occurs regardless of the type of drug involved, predominantly in women [183]. GSTT1 and/or GSTM1 null genotypes have been associated with hepatotoxicity triggered by tacrine, troglitazone [179,181] and anti-TB drugs [175,177,201,202]. Thus, these data support the role of GST in protection against hepatotoxicity.

On the other hand, depletion of free GSH due to GSH adducts formation has also been associated with DILI development [214].

#### 3.4.5. Transporters

Transport is the final step in determining the level of exposure to the reactive metabolite. One superfamily of proteins that has been proposed as a candidate for having a role in DILI susceptibility is the ATP-binding cassette (ABC) transporters [171]. They are implicated in the transport of bile acids and drugs. ABCB11 (encoding BSEP), ABCB4 (phospholipid flippase MDR3), and ABCC2 (bilirubin export pump MRP2) are the transporters most involved in DILI susceptibility. Mutations in these genes have been associated with higher risk of suffering cholestatic DILI due to impairment of bile secretion and accumulation of dangerous exogenous compounds (Table 2) [215]. Specifically, a study involving patients on treatment with a combination of anti-TB and antiretroviral therapy (ART) showed an association between the ABCB1 3435TT genotype (reported to have lower expression level and protein folding) and DILI development [216].

Moreover, genetic variants of ABCG2 are associated with hepatotoxicity induced by the tyrosine kinase inhibitor (TKI) sunitinib [217].

### 3.5. Antioxidant Defense System Genetic Polymorphisms

The magnitude of impact of reactive metabolites can be modified by a cellular response to oxidative stress that is generated. Polymorphisms in the genes which are involved in antioxidant defense processes may influence individual predisposition to DILI. The mutation (47T > C) of SOD2 results in an amino acid substitution (alanine for valine), leading to an increased import of SOD2 into the mitochondrial matrix [218] and finally to an augmented risk of developing cholestatic/mixed DILI [184]. Moreover, it has been suggested an association between SOD2 genotype and risk to develop hepatocellular DILI [182]. Interestingly, a specific genetic variant of Cu/Zn superoxide dismutase (SOD1) has been observed to be associated with a higher risk to develop DILI triggered by anti-TB drugs [219]. On the other hand, mutations in GPX1 linked with a reduced enzymatic activity have been also associated with DILI risk. GPX1 is the most abundant isoform of GPX proteins and catalyzes the reduction of H_2_O_2_ and other organic peroxides by oxidizing the reduced form of GSH. The polymorphism rs1050450 (198C > T) is the most studied one since it shows to reduce the enzyme activity by 40% [220,221]. Moreover, patients with the genetic variant GPX1 Pro200Leu show a higher risk of developing cholestatic DILI [184].

### 3.6. HLA Haplotypes

Multiple human leukocyte antigen (HLA) haplotypes are associated with increased risk of DILI development, suggesting a genetic predisposition to an adaptive immune response [165]. The associations identified in genome-wide genetic studies (GWAS) and candidate gene studies are generally drug-specific and involve MHC class I proteins (HLA-A, -B, -C) or MHC class II proteins (HLA-DP, -DQ, -DR) [222] (Table 3).

Currently, the strongest association observed between an HLA allele and DILI concerns HLA-B*57:01 and flucloxacillin [232,248,249]. However, because of the rarity of DILI associated with flucloxacillin, only 1:500–1000 patients carrying the risk allele will develop DILI [38], indicating that there must be other risk factors for flucloxacillin DILI apart from a specific HLA genotype and precluding the use of genetic testing in the pre-prescription of this antibiotic (very low positive predictive value [PPV]). However, HLA genotyping may be of value in reinforcing diagnoses due to the high negative predictive value (NPV). Conversely, HLA-B*57:01 association with abacavir treatment has an NPV of 100% and a PPV of 48% [250]. Thus, HLA-B*57:01 genotyping prior to abacavir prescription has been mandated by the FDA as well as the European Medicine Agency (EMA). Other HLA alleles with a strong DILI association are HLA DRB1*15:01-DQB1*06:02 haplotype for amoxicillin-clavulanic-associated hepatotoxicity [213,214,217]. Moreover, HLA-DRB1*07:01 carriers are at higher risk of developing DILI caused by ximelagatran [136] and lapatinib [237]. Interestingly, a recent GWA study identified a novel association between HLA-A*33:01 allele and DILI risk due to different drugs such as terbinafine, fenofibrate, and ticlopidine [231]. Moreover, the same study found an association between HLA-A*33:01 allele and cholestatic and mixed DILI, but not hepatocellular DILI, indicating that genetic factors also influence the DILI pattern.

It is important to notice that other liver disorders commonly mistaken with DILI are also related to HLA haplotypes. DILI can display autoimmune features mimicking idiopathic AIH [251]. Polymorphisms HLA-DRB1*03:01 and HLA-DRB1*04:01 are well-known susceptibility alleles for AIH development [252,253]. However, patients suffering from DILI with autoimmune features are not enriched in these alleles [254]. Hence, it would be important for the differential diagnosis to verify if a patient with suspected DILI carries these specific HLA alleles.

### 3.7. Other Genetic Polymorphisms Associated with DILI Susceptibility

Recently, the single nucleotide polymorphism rs2476601 (chr1: 114377568 > A/G) consisting of an amino acid change, tryptophan for arginine at codon 620 of the protein tyrosine phosphatase non-receptor type 22 (PTPN22) gene has been associated with DILI. PTPN22 function affects the responsiveness of T and B cell receptors, and different mutations of PTPN22 have been associated with susceptibility to autoimmune diseases [255]. A GWAS gathered a cohort of 2048 DILI cases and 12,429 control individuals where major ethnicities were included (European, African, American, and Hispanic) and showed a strong association of the rs2456601variant with DILI risk associated with many different classes of drugs [187]. This variant is also associated with autoimmune diseases risk, being linked with alterations in the composition of intestinal microbiota, reinforcing the role of immune system in DILI [256,257].

In summary, the study of genetic polymorphisms is a rapid technique to perform in patients, focused on genes involved in drug metabolism previously described, which have an association with DILI susceptibility [258]. However, the influence in clinical practice of polymorphisms involved in drug metabolism is over 20% to 25% of all current drug therapies, which support the hypothesis of DILI being a multifactorial disease [259], but also restricts the use of genetic testing in clinical practice due to its low PPV. However, HLA alleles are still a great contribution to improve the accuracy of DILI diagnosis [260] or to assist in distinguish DILI with autoimmune features from idiopathic AIH.

## 4. Biomarkers

Serum transaminases and bilirubin are the traditional biomarkers used for liver damage detection. Although useful, they have some limitations since they are not DILI-specific [261] (Table 4).

### 4.1. Diagnosis

Currently, serological biomarkers used in DILI diagnosis include ALT, aspartate aminotransferase (AST), ALP, and TBL [264].

At present, the only biomarker proposed for specific diagnosis of DILI is the protein-derived APAP-cysteine (APAP-CYS), useful for detecting APAP overdose [263]. However, it is difficult to develop specific biomarkers for DILI due to its low prevalence and its capacity of mimicking almost any hepatobiliary conditions. Some of the identified HLA risk alleles for DILI have an NPV > 95%, which enables the use of HLA genotyping to strengthen DILI diagnosis. In cases where the patient does not carry the allele of risk alternative etiologies should be searched out [272].

Glutamate dehydrogenase (GLDH) is a mitochondrial matrix enzyme required for acid metabolism, urea, and Krebs cycles. It shows the highest expression in liver tissue [273] and it has been used as a mechanistic biomarker of mitochondrial damage and for DILI outcome prediction. Due to the liver-specificity of GLDH, measuring its levels can differentiate liver from muscle injury, being a useful marker when ALT is elevated [267]. Although helpful, its sensitivity as diagnostic biomarker is poor, since there is controversy about its accuracy in predicting hepatocyte necrosis [167,264].

Very recently, Xiao et al. proposed advanced oxidation protein products (AOPPs) and ischemia-modified albumin (IMA) serum levels, as well as AOPPs/albumin (ALB) and IMA/ALB ratios as new oxidative stress biomarkers for DILI diagnosis and severity [262].

AOPPs are dityrosine-containing and crosslinking protein products formed during oxidative stress by reaction of plasma proteins with chlorinated oxidants, often carried by ALB in vivo [274]. Meanwhile, IMA is a known myocardial infarction biomarker that can be generated due to the modification of the N-terminus of ALB by ROS like superoxide and hydroxyl radicals [275].

A prospective, single-centered study was conducted by the screening of 128 patients with DILI (68 non-severe and 60 severe) and 38 healthy individuals [262]. AOPPs, AOPPs/ALB ratio, IMA, and IMA/ALB ratio were all positively correlated with ALP and TBL serum levels of DILI patients at admission. Moreover, DILI patients showed significantly higher AOPPs and IMA serum levels and AOPPs/ALB and IMA/ALB ratios as compared to those shown by controls. By performing a multivariate logistic regression model, Xiao et al. also concluded that patients with higher AOPPs and IMA serum levels were more likely to suffer from severe DILI. Meanwhile, patients with lower AOPPs and IMA serum levels were more likely to suffer from mild DILI. These results suggest these variables may be reliable new biomarkers to improve DILI diagnosis, although more studies with higher sample size are needed to confirm the association between AOPPs and IMA and DILI.

### 4.2. Prediction

Although many genetic polymorphisms have been associated with DILI (Table 2 and Table 3), almost none of them are being used as predictive biomarkers for different reasons. First, genetic polymorphisms found are normally associated with specific drugs, and their PPV is generally low. Nevertheless, it would be interesting to invest in predictive tests and use them in cases where drugs known to induce DILI are the only available treatment. Besides, it would be essential to determine which drug is causing liver injury when the patient is consuming different drugs with the same temporal pattern.

### 4.3. Prognosis

Most known prognosis biomarkers are serum biomarkers reported in APAP-DILI studies.

HMGB1 protein is usually associated with DNA under normal conditions, since it is involved in DNA replication, recombination, repair and gene transcriptional regulation. Upon damage, this protein is passively released by necrotic cells; therefore, it has been used as a necrosis and inflammation progression biomarker [23]. HMGB1 can also be secreted by monocytes and macrophages in a hyperacetylated form, acting as a late inflammation mediator [265]. Therefore, HMGB1 has been proposed as a promising biomarker in DILI due to its signaling role in inflammation and necrosis pathways. However, evaluation of HMGB1 in serum from patients is still needed and, until now, no studies have tested the prognostic value of HMGB1 in DILI. Moreover, the mechanisms by which HMGB1 may mediate injury progression, its role on systemic inflammation, the receptors involved and signaling pathways activated remain largely unknown [276]. In the same line, elevated levels of mtDNA were proposed as prognosis DILI biomarker, but it showed poor sensitivity values for DILI prediction [263].

Keratin-18 (K18) is a structural protein of the cytoskeleton that has a full-length form and a caspase-cleaved fragment (ccK18). Both forms are elevated in circulation after DILI, showing potential for prognostic use [167]. During hepatocellular necrosis, full-length K18 is passively released from necrotic cells into the blood. On the other hand, when apoptosis occurs, K18 is cleaved by caspases and released into the blood. Hence, early hepatocyte damage could be detected by measuring K18 (necrosis indicator) and ccK18 (apoptosis indicator) levels [266]. Despite the fact that serum proportion levels of K18 and ccK18 may be useful indicators of DILI, elevated levels of this protein are also found in patients with hypoxic hepatitis, alcohol steatohepatitis (ASH), non-alcoholic steatohepatitis (NASH), and other related disorders [263], in which may represent liver inflammation.

Macrophage colony-stimulating factor receptor (MCSFR), known as a marker of inflammation, is as a novel candidate biomarker for DILI, since it is believed to be released from activated macrophages during DILI [277]. In one study of Safer and Faster Evidence-based Translation (SAFE-T), patients under flupirtine treatment showed increased MCSFR levels in comparison to APAP-induced hepatotoxicity cases, suggesting that high serum/plasma levels of MCSFR may have value as a prognostic marker for liver disease associated with inflammation and immune system activation [32,267].

The other candidate soluble biomarker that was found to have prognostic ability in the study mentioned above was osteopontin (OPN), which has shown more predictive capacity than TBL [267]. OPN is an extracellular matrix phosphoglycoprotein that mediates diverse biological functions, such as cell-mediated immune responses, and plays a role in chronic inflammatory diseases [278]. Interestingly, elevations in serum and plasma OPN levels were also been found in different recent studies of ALF patients [279,280,281].

### 4.4. Extracellular Vesicles

The role of extracellular vesicles (EVs) as critical mediators of intercellular communication has become increasingly popular in the context of liver injury due to their implication for human diagnostics [282]. EVs are membrane-derived vesicles which can be released by different cell types to the extracellular media during liver injury, being found in biological fluids including blood and urine [283]. Regardless of the source cell, EVs carry lipids, proteins, coding, and non-coding RNAs and mtDNA. These vesicles can be captured through different mechanisms, being the most common the endocytosis of the vesicles and the subsequent release of the cargo inside the cytoplasm of the recipient cells, causing modifications in their physiological processes [284]. Therefore, many of the EVs-associated molecules such as proteins, mRNAs/microRNAs (miRNAs), and drug metabolizing enzymes (DMEs) could have potential efficacy as new biomarkers [285].

The molecules associated with EVs that are showing the most promising results as liver diseases biomarkers are the miRNAs [286]. miRNAs are small non-coding RNAs (19–22 nucleotides) which regulate gene expression. The mechanism underlying miRNAs relays on binding to 3′-untranslated sequence of mRNAs leading to their degradation or suppression of translation [287]. miRNAs can be found in two different ways: free circulating miRNAs or associated with vesicles [288]. EVs are generally well-preserved [289], thereby making EVs-miRNA stable biomarkers [290]. Changes in the expression of miRNAs are involved in different pathophysiological conditions, including liver injury [291] and it has been demonstrated that some miRNAs are associated with DILI development and progression. miR-122 is the most abundant miRNA in the liver [292]. Advantages of miR-122 as liver injury biomarker, include its higher liver-specificity, and greater sensitivity than ALT as it is detected earlier [268]. A recent study describing miRNA changes in sera of subjects with acute DILI showed that miR-122 was the most significantly elevated in DILI subjects compared to controls (20-fold approximately) [293]. Interestingly, miR-122 levels were lower among DILI subjects who died compared to those who survived, yet the same miRNA was significantly higher in DILI subjects who died than in controls. The authors hypothesized that, in the case of DILI survivors with higher miR-122, they could develop a compensatory response to liver injury that leads to recovery. Elevated levels of miR-122 have been also associated with APAP-DILI [294], drug-induced steatosis [295], and heparin treatment [296].

To characterize EVs released by drug-treated hepatocytes, Mosedale et al. used an in vitro model with primary human hepatocytes treated with tolvaptan. The authors found an increase in the release of EVs-miR-122 directly associated with mitochondrial-induced apoptosis [297]. The authors suggested that the release of EVs-miRNA, could also promote the adaptive immune response characteristic of DILI.

Interestingly, a recent work has demonstrated the role of EVs derived from healthy hepatocytes in maintaining normal liver immunotolerance, which can promote a tolerogenic immune state. On the contrary, changes of the EVs released by drug-treated hepatocytes can promote the loss of immune tolerance in the liver, since they are taken up by monocytes and deliver functional miRNAs and other contents acting DAMPs [298]. However, the lack of preclinical studies precludes the understanding of the function of EVs in DILI.

In summary, even though there are promising results, there are no specific biomarkers for DILI described to-date. Nonetheless, a combination of several biomarkers including miR-122, HGMB1, and total K-18, rather than an isolated analyte, could be of value as an early indicator of liver injury development.

## 5. Role of Oxidative Stress in DILI: Future Perspectives

There is a continuously growing knowledge of the contribution of oxidative stress and the antioxidant system in the underlying mechanism of DILI. Although DILI can arise from the concurrence of multiple factors or mechanisms, the oxidative stress-induced cell damage is highly consistent with other proposed mechanisms, given its links with mitochondrial damage, inflammation, immune response and cell death.

Drugs can induce oxidative stress through different mechanisms, such as generation of chemically reactive metabolites, impairment of the mitochondrial respiratory chain, depletion or reduction of the antioxidant enzymes pool, and induction of redox cycles [299]. Therefore, oxidative stress is thought to be the main mechanism implicated in the toxicity of many drugs, although the cell redox status imbalance leading to hepatocyte damage can be caused by different processes depending on the specific drug.

ROS production and antioxidant compounds depletion (e.g., GSH) are considered two of the most sensitive parameters of drug-induced hepatotoxicity [300,301,302]. Thus, future preclinical DILI studies will probably include high content screening assays in which oxidative stress would be an essential endpoint to measure [303,304,305,306].

Moreover, the wide number of associations between drug hypersensitivity reactions and polymorphisms of genes that encode enzymes related to the redox system suggests the important role of oxidative stress in onset and development of cell damage and tissue injury [307]. Individuals carrying specific polymorphisms in genes related to the cellular antioxidant mechanism and drug metabolism are more susceptible to DILI, suggesting that drug-induced oxidative stress involvement in DILI will also depend on host factors and will not have the same influence in all DILI cases.

Due the importance of oxidative stress in DILI, antioxidant therapy is thought to be a promising approach to prevent or manage DILI in the future. Although different randomized clinical trials have been conducted to assess the efficacy of various compounds with antioxidant properties [62], more high-quality clinical trials are needed to properly understand the effects of antioxidants in DILI.

The current research on diagnostic and prognostic biomarkers in DILI suggest that oxidative stress-related molecules could be reliable biomarkers in the future. As some examples, the abovementioned AOPPs/ALB and IMA/ALB ratios and GLDH levels seem to be promising candidates, although more studies with higher sample size are needed to confirm their relevance.

## 6. Conclusions

DILI represents a diagnostic challenge with increasing incidence over the last years. Ongoing prospective DILI registries and multinational collaborative efforts (European Cooperation in Science & Technology [COST] Action CA-17112, Prospective European Drug-Induced Liver Injury Network; https://proeurodilinet.eu/; accessed on 4 March 2021) are proving essential for a proper DILI characterization in phenotype and severity and for advancing knowledge on the mechanism underlying this disorder. This situation results from our lack of understanding of the pathophysiological mechanisms underlying the hepatotoxic reaction and the factors that contribute to the variability of the incidence, whether related to the host responses or to drug factors. Moreover, due to the multilayer nature of DILI, there are currently no functional animal models to study the underlying mechanism of the disease [308]. There are different mechanistic hypotheses about DILI development. It is clear that drug properties, host factors, and environmental conditions interact to determine DILI susceptibility, phenotypic expression, and outcome. It is possible that mitochondrial injury, oxidative stress, ER stress and/or inhibition of transporters are responsible for the upstream events of DILI or that these mechanisms are complementary and are involved in initiating an immune response. Since specific HLA genotypes are the genetic factor that more consistently have been associated with DILI risk, it seems that many DILI instances are, indeed, immune-mediated.

Based on clinical data from patients with idiosyncratic toxic drug reactions, different hypotheses on immune and non-immune-mediated mechanisms have been postulated to explain its mechanism of injury. Idiosyncratic DILI requires non-immune- and immune-mediated mechanisms for hepatic injury to occur. Usually, liver shows immunotolerance, since, due to its biological role, it is constantly exposed to foreign antigens. However, when the state of immune tolerance is broken, significant liver injury occurs [298,309].

The current lack of specific biomarkers often leads to incorrect diagnosis of DILI and inappropriate therapies in these patients. For that reason, further research on DILI biomarkers is urgently needed and in the meantime refinement of RUCAM scale, as well as its combination with others approaches including the new in vitro preclinical assays such as the MH cell test once it is properly validated, might be determinant for the achievement of an accurate DILI diagnosis.

Due to the relative rarity of DILI, pursuance in collaborative efforts reached during the last years is needed.

## Figures and Tables

**Table 1:** Drugs currently associated with drug-induced liver injury (DILI).

Drug	Indication	Type	Drug	Indication	Type
Abacavir	Antiretroviral	H/C	Allopurinol	Gout prophylaxis	H
Amiodarone	Anti-arrhythmic	H	Amodiaquine	Malaria treatment	H
Amoxicillin–clavulanic acid	Antibiotic	C/M	Angiotensin -converting enzyme inhibitors	Hypertension	C
Atorvastatin	Hypercholesterolemia	H/C	Azathioprine	Immunosuppressor	C
Bosentan	Hypertension	H/M	Carbamazepine	Anticonvulsant	H/C/M
Chlorpromazine	Antipsychotic	C/M	Clozapine	Antipsychotic	H/M
Cyclosporine A	Immunosuppressor	C	Dantrolene	Muscle relaxant	H
Diclofenac	Analgesic	H	Disulfiram	Alcoholism	H
Erythromycin	Antibiotic	C/M	Felbamate	Anticonvulsant	H
Fenofibrate	Hypertriglyceridemia and dyslipidemia	H	Floxuridine	Antineoplastic	H/C
Flucloxacillin	Antibiotic	C	Flupirtine	Analgesic	H
Flutamide	Nonsteroidal antiandrogen	H/C/M	Gabapentin	Anticonvulsant	C
Halothane	Anesthetic	H	Hydralazine	Antihypertensive	H
Ibuprofen	NSAID	H	Infliximab	Monoclonal antibody (Crohn’s disease, rheumatoid arthritis)	H
Isoniazid	Anti-tuberculotic	H	Ketoconazole	Fungicidal	H/C
Lamotrigine	Anticonvulsant	H	Lapatinib	Breast cancer	H
Leflunomide	Immunomodulatory agent	H/C	Lisinopril	Antihypertensive	H
Methotrexate	Antineoplastic	H	Methyldopa	Antihypertensive	H
Minocycline	Antibiotic	H	Nefazodone	Antidepressant	H
Nevirapine	Nonnucleoside reverse transcriptase inhibitor	C	Nitrofurantoin	Antibiotic	H/M
Pazopanib	Antitumor activity	M	Phenytoin	Anticonvulsant	H/M
Propylthiouracil	Antithyroid	H	Pyrazinamide	Anti-tuberculotic	H
Quinidine	Antiarrhythmic	C/M	Rifampicin	Anti-tuberculotic	H
Statins	Hypolipidemic	H/C	Sulfasalazine	Antirheumatic	M
Sulfonamides	Antibiotic	H/C	Sulindac	NSAID	H/C/M
Tamoxifen	Nonsteroidal antiestrogen	H/C/M	Terbinafine	Fungicidal	H/C
Thioguanine	Antitumor activity	M	Ticlopidine	Anti-platelet	C
TMP-SMX	Antibiotic	H	Tolcapone	Parkinson’s disease therapy	H
Tolvaptan	Hyponatremia treatment	H/M	Valproic acid	Anticonvulsant	H

H (hepatocellular), C (cholestatic), M (mixed). No data shown for withdrawn drugs. Updated information on the diagnosis, cause, frequency and patterns of liver injury induced by both prescription and non-prescription medications can be consulted in LiverTox (http://livertox.nlm.nih.gov, accessed on 8 January 2021). Moreover, categorization of drugs associated to DILI based on documented hepatotoxicity in the literature is available [22].

**Table 2:** List of genetic polymorphisms related to susceptibility of DILI development.

Genetic Variation	Association	Drug Studied	DILI Association	References
Drug transporters genes				
ABCB1 3435T	Transporter	Nevirapine	↓ Risk	[169,170]
ABCB11 1331C	Transporter	Various	↑ Risk	[171,172]
ABCB4 haplotypes	Transporter	Various	↑ Risk	[171]
ABCC2 haplotypes	Transporter	Various	↑ Risk	[164,173]
Cytochrome P450 genes				
CYP2B6*6	Phase I	Efavirenz	↑ Risk	[174]
		Nevirapine	↑ Risk	[175]
		Anti TBC	↓ Risk	[176]
CYP2B6 rs7254579	Phase I	Ticlopidine	↑ Risk	[177]
CYP2C8 haplotypes	Phase I	Diclofenac	↑ Risk	[164]
		Repaglinide	↑ Risk	[164]
CYP2E1 c1/c1	Phase I	Isoniazid	↑ Risk	[178]
Phase II enzymes genes				
GST T1/M1 null	Phase II	Various	↑ Risk	[179,180,181,182,183]
GPX1 198T	Phase II	Various	↑ Risk	[184]
NAT2 slow acetylators	Phase II	Anti-TBC	↑ Risk	[177,180,181]
SOD2 47C	Phase II	Various	↑ Risk	[182,184]
UGT2B7*2	Phase II	Diclofenac	↑ Risk	[164]
UGT1A6/1A9	Phase II	Tolcapone	↑ Risk	[185,186]
Others				
PTPN2	Tyrosine phosphatase	Various	↑ Risk	[187]
POLG	mtDNA polymerase γ	Valproic acid	↑ Risk	[100]

**Table 3:** Human leukocyte antigen (HLA) risk alleles associated with DILI susceptibility.

Drug	Genetic Variation	Odds Ratio	Population	References
Amoxicillin-clavulanate	A*02:01	2.2	Caucasian	[223]
	A*30:02	6.7	Caucasian	[224]
	B*18:01	2.9	Caucasian	[224]
	DRB1*07:01	0.18 ^	Caucasian	[225]
	DRB1*15:01-DQB1*06:02	3	Caucasian	[223,224,225,226,227]
Clometacin	B*08	-	Caucasian	[228]
Diclofenac	DRB1*13	^	Caucasian	[229]
Efavirenz + Anti-TB	B*57:02	8.1	African	[230]
	B*57:03	26.8	African	[230]
Fenofibrate	A*33:01	58.7	Caucasian	[231]
Flucloxacillin	B*57:01	80.6	Caucasian	[232]
	B*57:03	79.2	Caucasian	[233]
	DRB1*0701-DQB1*0303	9.7	Caucasian	[232]
Flupirtine	DRB1*16:01-DQB1*05:02	18.7	Caucasian	[234]
Lapatinib	DQA1*02:01	9–14.1	Caucasian	[235,236]
	DQB1*02:02	6.9–8.6	Caucasian	[235,236]
	DQB1*07:01	6.9–14.1	Caucasian	[235,236,237]
Lumiracoxib	DRB1*15:01-DQB1*06:02-DRB5*01:01-DQA1*01:02	5	Caucasian	[238]
Minocycline	B*35:02	29.6	Caucasian	[239]
Nevirapine	B*58:01	-	African	[240]
	DRB1*01:01	3–4.8	Caucasian	[241,242]
	DRB1*01:02	-	African	[240]
Pazopanib	B*57:01	2	-	[243]
Terbinafine	A*33:01	40.5	Caucasian	[231]
Ticlopidine	A*33:01	163.1	Caucasian	[231]
	A*33:03	13	Japanese	[244]
	B*44:03	6.6	Japanese	[244]
	Cw*1403	7.3	Japanese	[244]
	DQB1*06:04	10.1	Japanese	[244]
	DRB1*13:02	9	Japanese	[244]
Tiopronin	A*33	-	Japanese	[245]
Trimethoprim-Sulfamethoxazole	B*14:01	9.2	Caucasian	[246]
	B*35:01	-	Africans	[246]
Ximelagatran	DRB1*07:01-DQA1*02	4.4	Caucasian	[136]
	DQB1*02:01	-	Indian	[247]

^ protective effect.

**Table 4:** Serum biomarkers associated with DILI.

Serum Biomarkers	Advantage	Limitations	Comments	References
AOPPs, IMA	- Correlation with ALP, TBL - Diagnosis and severity	- Not DILI-specific - Not liver-specific - Not prognostic biomarkers	- Associated with oxidative stress.	[262]
APAP-CYS	- Specific for APAP overdose - Diagnosis - Sensitive and specific	- Only valid for diagnosis of APAP-induced liver injury.	- Three approaches to measure it: Immunoassay HPLC-EC HPLC-mass spectrometry	[263]
Bilirubin	- Liver-specific - Elevations of TBL levels correlate with whole liver function.	- Not DILI-specific - Do not provide information regarding the mechanism of the injury.	- Identify potential DILI cases after DILI injury has occurred.	[261,264]
GLDH	- Liver-specific - Not altered in muscle injury - Not impacted by gender or age - Diagnosis	- Poor sensitivity - Not DILI-specific	- Associated with mitochondrial damage	[161,255,256]
HMGB1	- Necrotic and inflammation marker - Prognosis	- Not liver-specific - Not DILI-specific	- High levels are associated with poor outcome	[23,265]
K18	- Ratio ccK18:K18 predicts degree of injury and cell death type - Prognosis	- Not liver-specific - Not DILI-specific	- K18 determines likelihood of poor outcomes	[266,267]
MCSFR	- Indicative of severe DILI - Inflammation marker - Prognosis	- Not liver-specific	- Controls macrophages proliferation, differentiation and function	[267]
miR-122	- Early diagnosis and prognosis	- High variability among individuals - Not DILI-specific	- Associated with mitochondrial damage - Detected free circulating or in EVs	[268,269]
mtDNA	- Useful for prognosis - Mechanistic biomarker - Early prediction for acute injury	- Not DILI-specific - Not hepatocellular damage-specific - Poor sensitivity	- Associated with mitochondrial damage	[167,270]
OPN	- Adaptation/repair/survival biomarker - Prognosis biomarker	- Not liver-specific - Not DILI-specific	- Pro-inflammatory cytokine - Associated with necrosis levels	[264,267]
Transaminases	- Can reflect hepatic lesions - Diagnosis and prognosis	- Not liver-specific - Not DILI-specific	- Associated with muscle and cardiac damage - Poor correlation with histological patterns and lesion severity	[23,271]

AOPPs, advanced oxidation protein products; APAP-CYS, acetaminophen-cysteine; GLDH, glutamate dehydrogenase; HMGB1, high mobility group box protein 1; HPLC-EC, High Performance Liquid Chromatography with Electrochemical Detection; IMA, ischemia-modified albumin; K18, keratin-18; ccK18, caspase-cleaved keratin-18; MCSFR, macrophage colony-stimulating factor receptor; miR-122, microRNA-122; mtDNA, mitochondrial DNA; OPN, osteopontin; TBL, total bilirubin.

## Data Availability

Not applicable.

## Abbreviations

ABC, ATP-binding cassette; AIH, autoimmune hepatitis; ALB, albumin; ALF, acute liver failure; ALP, alkaline phosphatase; ALT, alanine aminotransferase; AOPPs, advanced oxidation protein products; APAP, acetaminophen; APAP-CYS, APAP-cysteine; APCs, antigen-presenting cells; ARE, antioxidant response element; ART, antiretroviral therapy; ASH, alcohol steatohepatitis; ASK1, apoptosis signal regulating kinase 1; AST, aspartate aminotransferase; ATF6, activating transcription factor 6; ATP, adenosine triphosphate; BiP, binding immunoglobulin protein; BSEP, bile salt export pump; CAR, constitutive androstane receptor; ccK18, caspase-cleaved K18; CIOMS, Council for International Organizations of Medical Sciences; CNS, central nervous system; COST, Cooperation in Science & Technology; CYP450, cytochrome P450; DAMPs, damage-associated molecular patterns; DILI, drug-induced liver injury; DILIN, drug-induced liver injury network; EASL, European Association for the Study of the Liver; EMA, European Medicine Agency; ER, endoplasmic reticulum; EVs, extracellular vesicles; ETC, electron transport chain; FasL, Fas ligand; FDA, Food and Drug Administration; γ-GT, γ-glutamyl transpeptidase; GCLC, glutamate-cysteine ligase catalytic subunit; GLDH, glutamate dehydrogenase; GPX, glutathione peroxidase; GSH, glutathione; GSK-3β, glycogen synthase kinase-3b; GST, glutathione S-transferase; GWAS, genome-wide association study; HDS, herbal and dietary supplements; HILI, herbal-induced liver injury; HLA, human leukocyte antigen; HMGB1, high mobility group box protein 1; HO-1, heme oxigenase-1; HPLC, high performance liquid chromatography; HPLC-EC, HPLC with Electrochemical Detection; HSP, heat shock proteins; IHN, isoniazid; IFNγ, interferon gamma; IMA, ischemia-modified albumin; IRE1, inositol-requiring enzyme 1; JNK, c-Jun N-terminal kinases; Keap1, Kelch-like ECH-associated protein 1; K18, keratin-18; LPO, lipid peroxidation; MCSFR, macrophage colony-stimulating factor receptor; MDA, malondialdehyde; MgIG, magnesium isoglycyrrhinate; MH, monocyte-derived hepatocyte-like; MHC, major histocompatibility complex; miRNA, microRNA; MLK3, mixed-lineage kinase-3; MPT, mitochondrial permeability transition; MRP, multidrug resistance-associated proteins; mtDNA, mitochondrial DNA; MTX, methotrexate; NAC, N-acetylcysteine; NAPQI, N-acetyl-p-benzo-quinone imine; NASH, non-alcoholic steatohepatitis; NAT, *N-*acetyl transferases; NPV, negative predictive value; NQO1, NAD(P)H quinone oxidoreductase 1; Nrf2, nuclear factor erythroid 2-related factor 2; NSAIDs, nonsteroidal anti-inflammatory drug; OPN, osteopontin; OXPHOS, oxidative phosphorylation; PERK, protein kinase RNA-like ER kinase; p-i, pharmacological interaction; PRRs, pattern-recognition receptors; PPV, positive predictive value; PRS, polygenic risk score; PTPN22, protein tyrosine phosphatase non-receptor type 22; PUFAs, polyunsaturated fatty acids; PXR, pregnane X receptor; ROS, reactive oxygen species; RUCAM, Roussel Uclaf Causality Assessment Method; SAFE-T, Safer and Faster Evidence-based Translation; SOD1, Cu/Zn superoxide dismutase; SOD2, superoxide dismutase 2; TB, tuberculosis; TBL, total bilirubin; TCR, T cell receptor; TKI, tyrosine kinase inhibitor; TLR, Toll-like receptor; TMP-SMX, Trimethoprim-Sulfamethoxazole; TNFɑ, tumor necrosis factor-alpha; TRAIL, TNF-related apoptosis-inducing ligand; UGT, UDP-glucuronosyltransferases; ULN, upper limit of normal; UPR, unfolded protein response; VPA, valproic acid.
